# So. Med. College Commencement, &c.

**Published:** 1889-04

**Authors:** 


SOUTHERN MEDICAL COLLEGE COMMENCEMENT.—The Commencement Exercises of the Southern Medical College took place on the evening of the 2d of March, at De Gives' Opera House, in Atlanta.

The evening was exceedingly wet and inclement, and yet there was a large audience in attendance.

After prayer, by the Rev. Mr. Walker, Dr. Wm. Perrin Nicolson, the dean, made his annual report of the condition of the Institution, which was in all respects a very encouraging one—the school having steadily progressed from its origin until the present time. It now possesses advantages and facilities for imparting a medical education second to no school in the Union. Only two years ago a Dental Department was instituted, which also is thoroughly equipped, having an able faculty and all the needed apparatus for imparting the most thorough dental instruction.

After the report of the Dean diplomas were conferred upon the following graduates in medicine and dentistry by Professor T. S. Howell, the President of the College, to-wit—*In Medicine:* T. C. Avary, Georgia; J. T. Barnwell, Georgia; W. R. Belcher, Alabama; T. N. Bingham, Georgia; C. A. Blount, North Carolina; R. L. Boynton, Georgia; W. C. Bryant, Georgia; W. W. Carmical, Georgia; E. H. Cook, Maine; W. H. Coston, Alabama; J. A. Craven, North Carolina; D. M. Davis, Alabama; J. J. Foster, Georgia; W. T. Haygood, Georgia; E. J. Hawkins, Alabama; J. L. Hilt, Alabama; W. A. Hitchcock, Georgia; W. E Holtzclaw, South Carolina; S. W. Jackson, Alabama; R. A. Justice, Georgia; A. P. Kemp, Georgia; J. R. Kinney, Georgia; W. C. Kiser, North Carolina; B. B. Lancaster, North Carolina; J. W. Nelms, Georgia; W. W. Rutland, Alabama; B. D. Smith, Georgia; R. W. Trotter, Georgia; T. W. Tucker, Georgia; M. M. Warren, Mississippi; J. C. White, Georgia; W. M. Wilson, Georgia; J. H. Winchester, Georgia; W. T. Wise, Alabama. *In Dentistry*—J. A. Arbeely, Damascus, Syria; Aaron Brauch, Georgia; M. Z. Crist, Kentucky; O. H. Cantrell, Georgia; F. W. Daniel, Louisiana; F. W. Duke, Georgia; C. W. Forehand, Georgia; H. J. Gasland, Georgia; T. M. Hyman, Georgia; J. A. Link, Georgia; R. L. Lane, Alabama: S. W. Lide, Georgia; B. R. McBath, Tennessee; T. B. Pilcher, Georgia; R. G. Ragan, Georgia; W. T. Sinclair, North Carolina; H. R. Williamson, Georgia.

After the conferring of the degrees, President Powell made an address to the graduating class, which was eminently able and appropriate—at a future time we will publish the address in this JOURNAL.

It was followed by an excellent and eloquent valedictory from Dr. S. W. Jackson, of Alabama, representing the medical class. His subject was *Labor Omnia Vincit* It was ably discussed, gracefully delivered, and well received.

Following this address was that of the valedictorian of the Dental class—B. R. McBath of Tennessee—his subject, *The Glory of the New South*, was ably treated and admirably delivered. The two young valedcitorians acquitted themselves with honor, and each received floral tokens of approval from ladies in the audience.

The annual oration was delivered by the Rev. H. K. Walker, of Marietta, Ga. Mr. Walker is a young man of fine attainments and of marked ability. His address was a very able and interesting one, and was well and gracefully delivered. It made a profound impression and was greeted with frequent applause. So pleased were we with the address that a copy was procured for publication, and may be found in the present number of this JOURNAL.

*The Prizes* conferred upon the graduates in medicine are as follows:

First Honor—By the Faculty—To John T. Barnwell, of Georgia.

Second Honor—by the Faculty—To W. Wise, of Alabama.

Prize in Obstetrics—By Prof. T. S. Powell—To John C. White, of Georgia.

Prize in Physiology—By Prof. R. C. Word—To W. E. Holtzclaw, of South Carolina.

Prize in Anatomy—By Prof. W. P. Nicolson—To B. D. Smith, of Georgia.

Prize in Surgery—By Prof. J. M. F. Gaston—To M. M. Warren, of Georgia.

Prize in Ear, Eye and Throat—By Prof. A. G. Hobbs—To W. O. Hitchcock, of Georgia.

Prize in Practice—By Prof. W. D. Bizzell—To T. W. Tucker, of Georgia.

Prize in Materia Medica—By Prof. G. Roy—To T. C. Avary, of Georgia.

Prize in Chemistry—By Prof. Burns—To E. H. Cook, of Maine.

*Of the Dental Department*, Prof. Carpenter, the Dean, gave a most gratifying report. There was in attendance at this, the second term, thirty-six students and seventeen graduates—coming from Iowa, Kentucky, Tennessee, North Carolina, Georgia, Alabama, Louisiana, Virginia and from Syria—the Old World. Prof. C. Spoke in high terms of the intelligence of the present class, and of the great proficiency of the graduates, as shown in their examinations and in the practical exhibit of actual work performed by them. He also spoke in most complimentary terms of the faculty, and of the splendid equipment and facilities furnished by the school for the most thorough instruction in dentistry.

The following are the prizes presented to the successful contestants in dentistry:

First Honor—By the Faculty—To M Z Crist, of Kentucky.

Prize in Pathology and Therapeutics—By Prof Carpenter—To H J Garland, of Georgia.

Prize in Operative and Clinical Dentistry—By Prof Wm Crenshaw—To W T Sinclair, of North Carolina.

Prize in Oral Surgery and Materia Medica—By Prof R Y Henley—To B R McBath, of Tennessee.

Prize in Mechanical and Prosthetic Dentistry—By Prof J S Thompson—To Aaron Brauch, of Georgia.

Prize in Chemistry and Metallurgy—By Prof S G Holland—To J A Link, of Georgia.

Prize in Physiology—By Prof R C Word—To W T Sinclair, of North Carolina.

Prize in Anatomy—By Prof W P Nicolson—To J A Link, of Georgia.

Prize from Demonstrator Dr Thomas Crenshaw, to W T Sinclair, of North Carolina.

Prize for Best Gold Filling—By Holliday Bros—To J W Daniel, of Georgia.

Prize for Best Junior Examination—By the Faculty—To E G Thomas, of Georgia.

There was delightful music between the several parts of the programme. The lights were brilliant, the stage decorations beautiful, and the exercises were greatly enlivened by the presence of the ladies, who made glad the hearts of the graduates by a profusion of floral offerings. Altogether, it was among the most attractive occasions of the kind ever witnessed in Atlanta.

